# Folded Fascia Lata Allograft Reinforcement for Recurrent Postsurgical Synovial Fistula of the Knee: A Case Report

**DOI:** 10.7759/cureus.116207

**Published:** 2026-09-14

**Authors:** Julio Cesar Gali, David Furtado Cavalcante Vecina, Erik Vecina, Luis Amancio Gomes, Marco Antônio Camarão Pinheiro

**Affiliations:** 1 Surgery, Pontifícia Universidade Católica de São Paulo, Sorocaba, BRA; 2 Surgery, Corts Clínica de Ortopedia, Reabilitação e Traumatologia, Sorocaba, BRA

**Keywords:** case report, fascia lata allograft, knee, surgery, synovial fistula

## Abstract

Synovial fistulas of the knee are a rare complication of surgical procedures and are often underreported because they usually do not cause significant deleterious effects. Arthroscopy-related fistulas are generally managed conservatively, while persistent fistulas following open procedures can be challenging to treat and may require further surgical intervention. We report the case of a patient with a recurrent knee synovial fistula following seven previous surgical procedures. Despite multiple treatment attempts, the fistula persisted. Definitive treatment was performed using reinforcement of the fistulous tract with a folded fascia lata allograft. Postoperatively, the fistula healed completely, and the patient was able to return to sports activities without recurrence. Folded fascia lata allograft reinforcement may be an effective treatment option for recurrent knee synovial fistulas when conventional surgical techniques have failed. This technique achieved successful fistula closure and allowed return to sports activity in the patient presented.

## Introduction

The knee capsule normally forms a sealed compartment that retains synovial fluid, which lubricates the joint and nourishes the articular cartilage. A synovial fistula is an abnormal communication between the joint capsule and the skin or an adjacent soft-tissue space, through which synovial fluid continuously leaks [1]. This complication may arise after arthroscopic procedures or open surgery. Fistulas may drain externally through the skin or internally into adjacent structures, such as the prepatellar bursa [2]. Continuous production and extravasation of synovial fluid lead to persistence of the fistula [3], with increased drainage typically observed during knee flexion [1]. Beyond local discomfort and impaired wound healing, an untreated or mismanaged fistula carries the risk of retrograde infection of the joint, which is why prompt and appropriate management matters [1].

The occurrence of fistulas after arthroscopy is rare. DeLee reported 980 complications in a total of 118,590 arthroscopies, with 30 cases of synovial fistula (3.2% of all complications) [4]. Proffer et al. evaluated 976 arthroscopic knee surgeries, and six knees in six patients presented with synovial fistula (0.61%) [5]. Kim et al. reported one case of synovial fistula in 40 consecutive patients who underwent arthroscopic partial meniscectomy through an inframeniscal portal [6]. Yiannakopoulos reported four cases of synovial fistula of the knee that occurred after approximately 2,300 arthroscopies (0.17%) [1]. Bohensky et al. quantified the frequency of adverse outcomes after elective knee arthroscopies in Australia: among 180,717 elective knee arthroscopies in 150,490 patients, with 2,483 readmissions due to complications, three (1.0%) were synovial fistulas [7].

This complication is likely underreported due to its generally benign clinical course [1]. Proffer et al. suggested that pathological changes in the articular cartilage and the use of a posterior portal may be risk factors for the development of fistulas [5]. Conservative treatment is usually effective and consists of immobilization with the knee in extension for one to two weeks. If immobilization does not promote healing, surgical excision of the fistula is recommended [1]. Yiannakopoulos specifically warns against suturing or simply observing a fistula, since either can lead to knee infection instead of resolution [1]. However, these conventional strategies assume a single, well-defined fistulous tract that can be excised or sealed at one point. Therefore, they may be insufficient when the underlying defect is diffuse rather than focal; for example, when prior soft-tissue procedures around the joint have caused generalized capsuloligamentous weakening rather than a localized communication. Reconstructive alternatives described for refractory or complex fistulas rely mainly on flap-based techniques, such as myofascial flaps [8] or free fasciocutaneous flaps [9], or on a one-way valve mechanism created with autologous fascia lata at a single point of closure [10]; these approaches, while effective, either require the morbidity of a vascularized tissue transfer or remain designed for a localized, single-point defect. To our knowledge, the use of a folded fascia lata allograft to provide extensive structural reinforcement over a diffusely compromised area, rather than the focal closure of a single tract, has not been previously described for recurrent post-arthroscopic synovial fistula of the knee; furthermore, this technique avoids donor-site morbidity by using allograft rather than autologous tissue.

We report a case of a knee fistula that recurred after seven previous surgeries, successfully treated with a folded fascia lata allograft. This report aims to describe the surgical technique in detail and to discuss its potential as a reconstructive alternative for recurrent synovial fistulas refractory to conventional treatment.

## Case presentation

A 37-year-old male recreational runner with no relevant comorbidities, no prior knee history, and no identifiable risk factors for impaired wound healing underwent arthroscopic removal of a symptomatic synovial plica and pie-crusting of the iliotibial band in June 2018.

In December 2018 (six months later), recurrent symptoms prompted a second arthroscopic procedure, consisting of repeat plica excision, surgical lengthening of the iliotibial band, and osteotomy of the lateral epicondyle.

In January 2019, the patient underwent drainage of the surgical wound. Cytochemical analysis of the synovial fluid revealed a leukocyte count of 71,680 cells/µL (reference: ≤200 cells/µL), and culture was positive for *Streptococcus agalactiae*; white blood cell count and inflammatory markers were within normal limits at this time and remained so throughout subsequent follow-up. The patient was treated with cefuroxime and ciprofloxacin for seven days. Ten days later, a fistula was identified, and surgical debridement and irrigation of the infectious focus were performed, followed by six weeks of intravenous daptomycin and ceftriaxone.

In April 2019, an MRI scan demonstrated a fistulous tract extending from the lateral skin surface intra-articularly (Figure 1). Five days later, a further debridement procedure was performed, but the fistula itself was not identified intraoperatively. Between April and October 2019, eight subcutaneous infiltrations of triamcinolone acetonide 20 mg were administered at another institution, without resolution.

**Figure 1:**
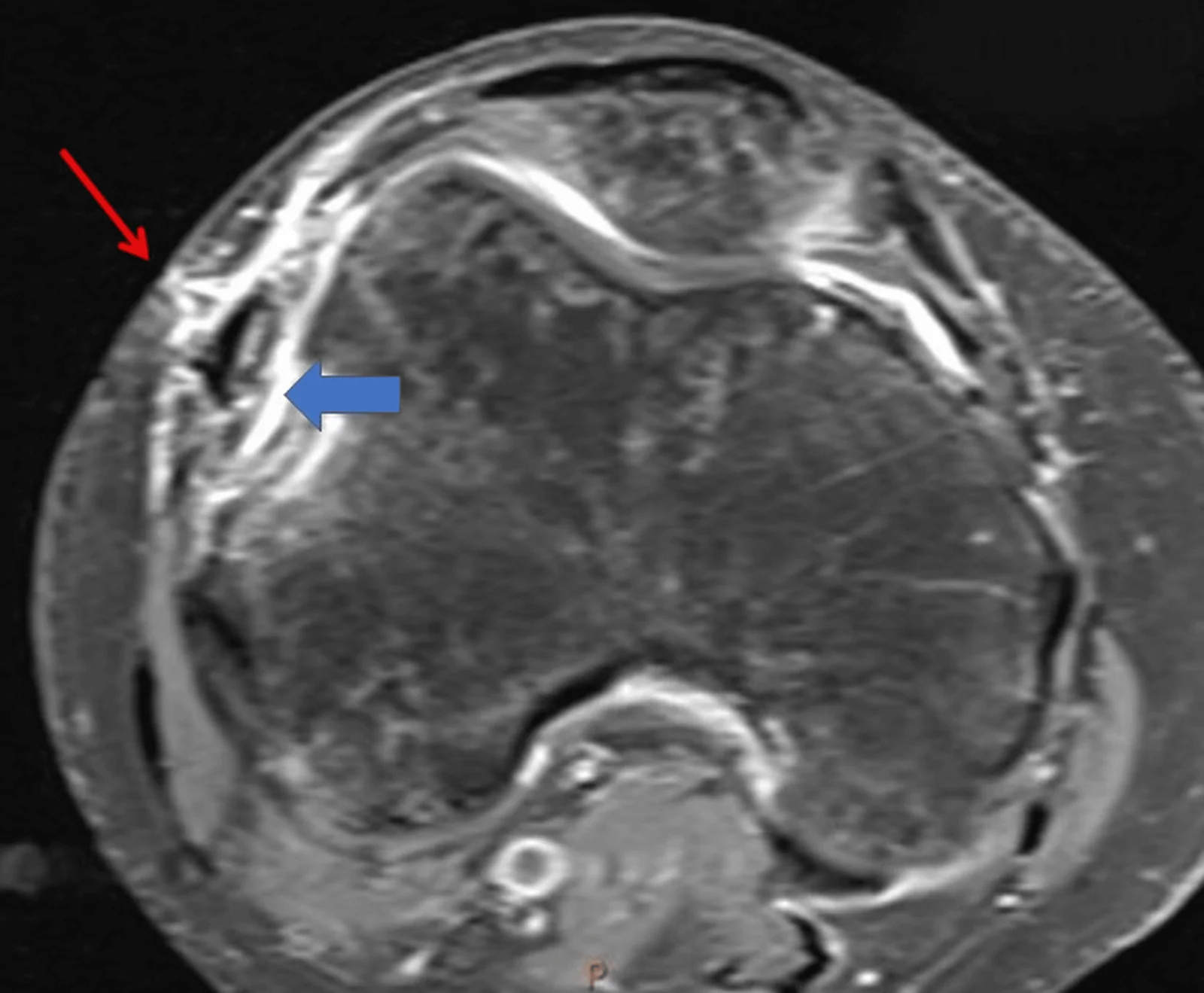
Axial contrast-enhanced, fat-suppressed T1-weighted MRI of the right knee at the level of the lateral femoral condyle, showing a fistulous tract extending from the lateral cutaneous surface of the knee (red arrow) to the intra-articular compartment (blue arrow).

In January 2020, the patient presented with a new episode of purulent drainage. Cytochemical analysis of the synovial fluid at this time showed a leukocyte count of 111,786 cells/µL (reference: ≤200 cells/µL). MRI performed in February 2020 showed thin fluid collections between the subcutaneous tissue and the lateral ligament complex and lateral femoral condyle, with small capsuloligamentous openings connecting these compartments, suggestive of intra-articular extension; no discrete fistula was demonstrated on this study (Figure 2). In March 2020, soft tissue and bone specimens were surgically collected to rule out infection by slow-growing bacteria or fungi; osteomyelitis was excluded, but persistent drainage continued postoperatively. Twenty sessions of hyperbaric oxygen therapy were subsequently performed at another institution, again without resolution.

**Figure 2:**
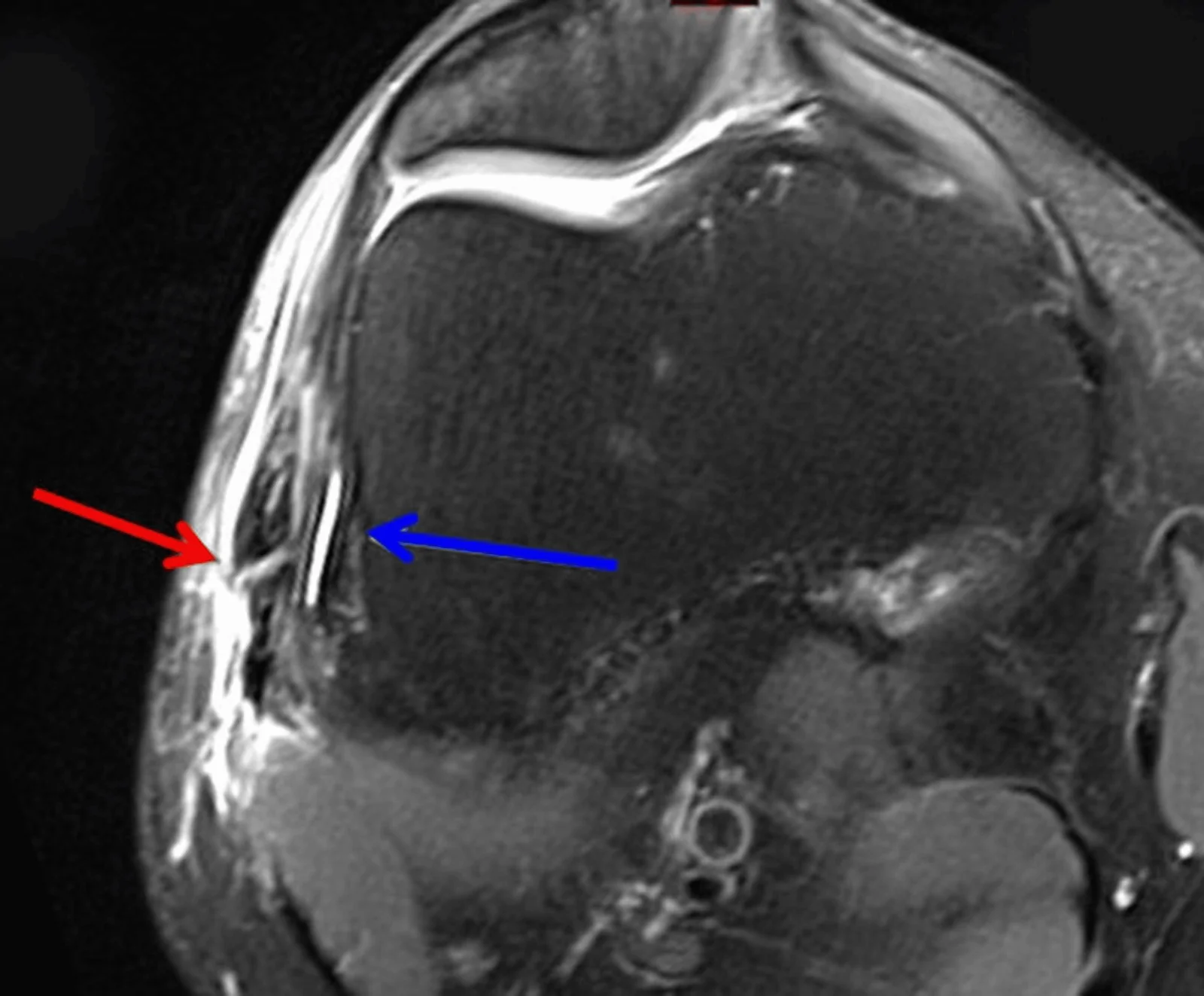
Axial contrast-enhanced, fat-suppressed T1-weighted MRI of the right knee, showing thin fluid collections between the subcutaneous tissue and the lateral ligament complex (red arrow), and between the lateral ligament complex and the lateral femoral condyle (blue arrow), with small capsuloligamentous defects interconnecting these two compartments.

In June 2020, a plastic surgeon performed debridement and closure of the articular fistula; the same procedure was repeated 15 days later, also without success. The patient was then referred to our service, presenting with an intra-articular suction drain in place and continuous drainage of fluid consistent with synovial fluid. Drainage was consistently exacerbated by knee flexion.

In July 2020, based on the history of iliotibial band lengthening and lateral epicondyle osteotomy together with the most recent MRI findings, a different surgical strategy was adopted. On examination under anesthesia, full extension was achieved, with flexion limited to 100°; mild erythema and edema were noted over the previous surgical incision. With the knee flexed at 90°, an incision was made through the previous scar, exposing the fascia lata and the lateral epicondyle. Methylene blue was injected intra-articularly and extravasated through multiple sites in the fascia lata, which appeared diffusely thinned and attenuated; no single discrete fistulous tract was identified intraoperatively. Given the absence of a localized opening to excise or seal, a structural reinforcement strategy was chosen over point closure, aiming to create a protective seal over the weakened fascia while avoiding a rotational flap.

A folded fascia lata allograft measuring 9 cm in width and 4 cm in height, obtained from a screened donor and processed by chemical cleansing with antibiotic solutions and low-dose gamma irradiation, then cryopreserved until use, was centered over the lateral epicondyle. Its anterior portion was sutured to the patient's native fascia, and fibrin glue was applied to the portion of the graft in contact with the iliotibial tract; the posterior portion was then sutured, followed sequentially by the distal and proximal portions (Figure 3).

**Figure 3:**
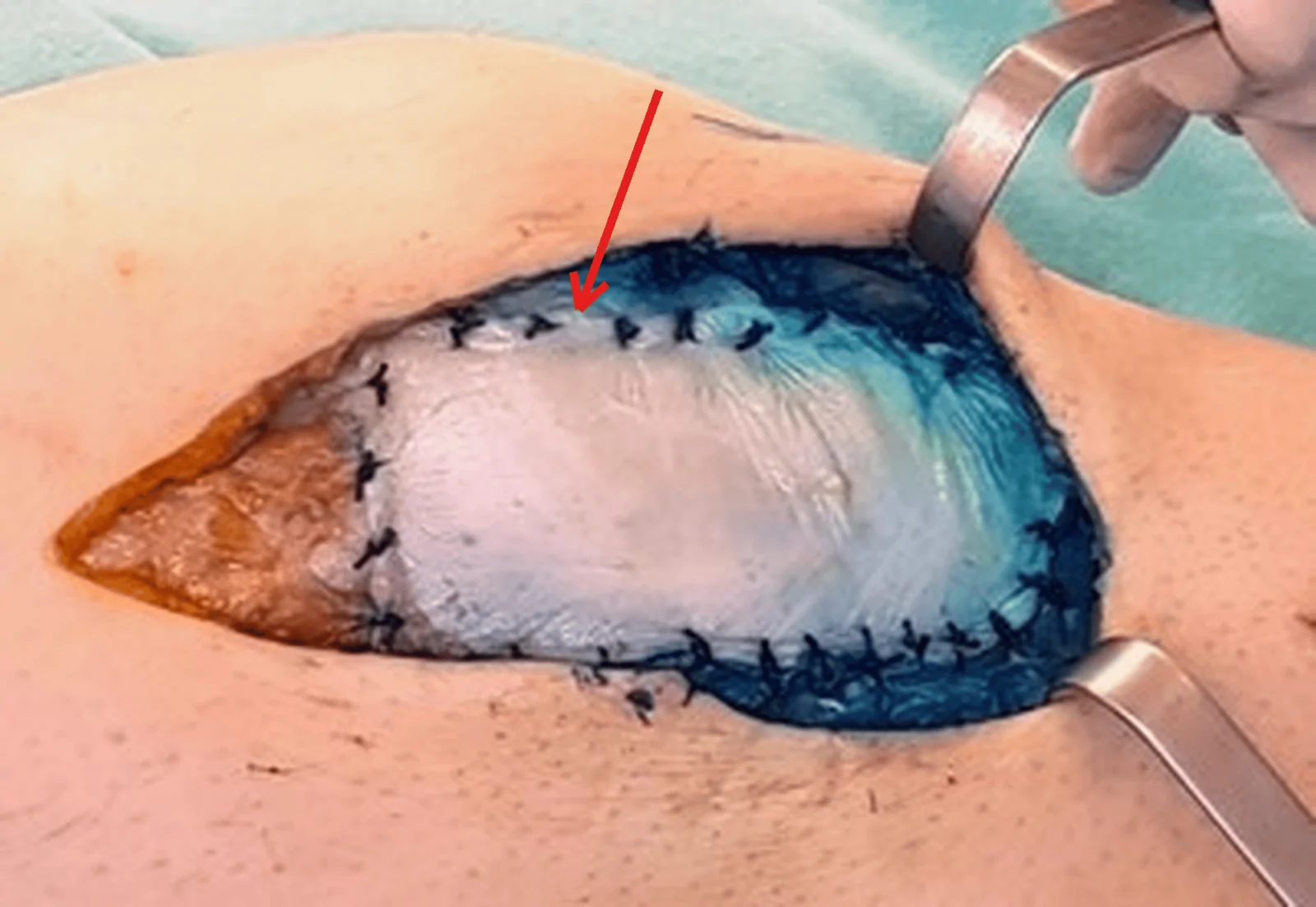
Intraoperative lateral view of the right knee showing the folded fascia lata allograft (red arrow) sutured over the patient's iliotibial tract, prior to skin closure.

The patient was discharged on the first postoperative day without immobilization and was allowed to bear weight as tolerated, using crutches for seven days. Straight-leg raises, knee flexion, and isometric quadriceps exercises were initiated on postoperative day 1. By the fourth postoperative week, walking, stationary cycling, and swimming were introduced; gym-based strengthening began at two months, and running was permitted at three months. The fistula closed completely, and MRI performed in November 2020 showed fibrotic thickening of the distal iliotibial tract, with reduction of edema and fluid distension of the adjacent soft tissues (Figure 4). The patient returned to sports six months postoperatively.

**Figure 4:**
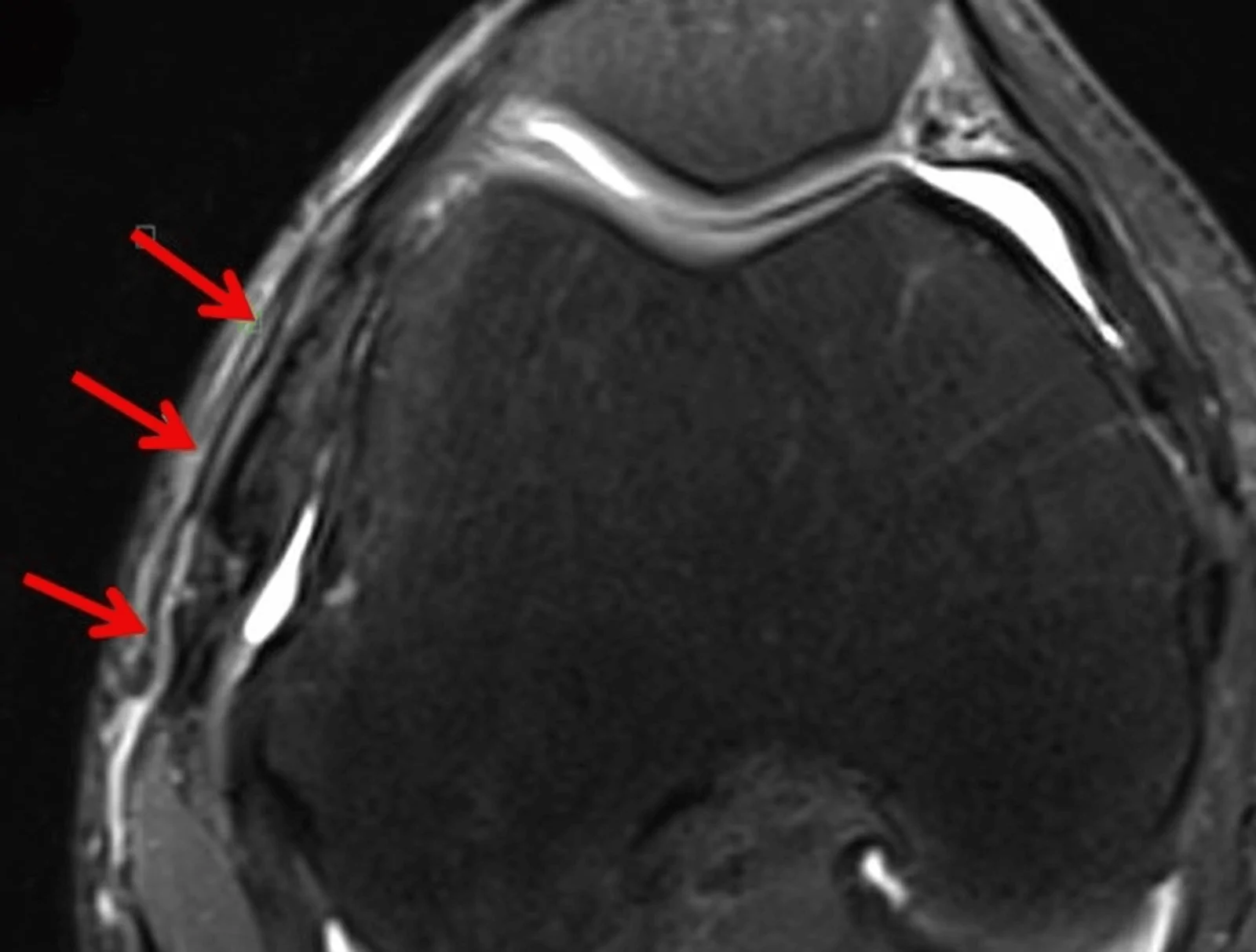
Axial contrast-enhanced, fat-suppressed T1-weighted MRI of the right knee obtained four months postoperatively, showing fibrotic thickening of the distal iliotibial tract (red arrows) and reduction of edema and fluid distension of the adjacent soft tissues, compared with preoperative imaging.

At nearly six years of follow-up, the patient remained pain-free, with full extension and 130° of flexion of the operated knee. He presented only mild residual swelling over the distal and lateral aspect of the operated thigh and had returned to his preferred sport, long-distance running.

## Discussion

Synovial fistula of the knee may arise after arthroscopic procedures or open surgeries. These fistulas may drain externally through the skin [1] or internally into the knee, such as into the prepatellar bursa [2]. In the presence of a fistula resulting from an arthroscopic procedure, DeLee stated that excision or immobilization is required [4]. Proffer et al. [5] recommended closure of the arthroscopy portals with sutures and immobilization of the knee in extension for seven to 14 days; furthermore, if the obtained cultures are negative, prophylactic antibiotic therapy would not be recommended.

On the other hand, Yiannakopoulos [1] treated four patients with knee fistulas following arthroscopic procedures using closure of all portals with sutures, immobilization in extension for one to two weeks, and antibiotic therapy. This approach was successful in three patients; in the remaining patient, the fistula was excised, and the synovium and knee capsule were repaired due to failure of conservative treatment. Odumala et al. [11] reported a case of fistula following knee arthroscopy for removal of loose bodies in a man with osteoarthritis. The fistula resolved after repeat knee arthroscopy, irrigation, and six weeks of antibiotics for treatment of a staphylococcal infection.

Some authors have reported the development of fistulas following trauma and open surgical procedures, especially those involving the use of orthopedic fixation material. Manocha [10] reported that a 17-year-old male patient sustained a penetrating injury to the right knee following a motorcycle accident, resulting in a displaced osteochondral fragment from the patellofemoral joint. The wound was initially treated with primary closure and immobilization.

Nineteen weeks later, the patient developed a persistent fistula with watery drainage associated with herniation of synovial tissue. MRI revealed a tear of the lateral retinaculum and a fistulous tract communicating with the joint cavity. During surgery, a large lateral capsular defect unsuitable for direct closure was identified. An autologous fascia lata graft was then used and sutured in a double-fold configuration to create a valve mechanism capable of preventing joint fluid leakage through the suture line. After three years, the patient remained asymptomatic, with no clinical signs of residual synovitis, and MRI demonstrated preservation of the valvular mechanism with adequate watertight closure.

van Kampen and Hinten [3] published three cases of knee fistula. In the first case, the fistula developed after a tibial biopsy and was resolved by filling the defect with autologous cancellous bone graft. The second case occurred in a patient who underwent a high tibial osteotomy, and treatment consisted of knee immobilization for six weeks. In the third patient, the fistula developed after anterior cruciate ligament reconstruction, in which signs of intraosseous fluid were present in the lateral condyle. The fistula healed after the defect was filled with polymethylmethacrylate bone cement.

Marberry and Ginsburg [12] reported a case of fluid accumulation in the prepatellar space communicating with the knee joint through the cannulated portion of a screw, eight weeks after open reduction and internal fixation of an osteochondral patellar fracture. The patient was treated with symptomatic medication, ice application, and a compressive dressing. Momaya et al. [13] described a synovial fistula that developed after removal of a tension band plate, irrigation and debridement, and a split-thickness skin graft. The fistula healed with cast immobilization of the knee in extension for six weeks. Hadied [2] reported a fistula between the knee and the prepatellar bursa following arthroscopic lateral retinacular release. The edges of the bursa were freshened and sutured, while the lateral retinaculum was left open. The patient also used a cast for six weeks.

More recently, Boushnak et al. [14] reported a case of fistula arising from an anteromedial wound at the tibial tunnel site two years after anterior cruciate ligament reconstruction using a graft with suspensory fixation on both the femoral and tibial sides. The condition was successfully treated with tunnel debridement, bone grafting, placement of a bioresorbable interference screw, and cement augmentation to achieve complete tunnel sealing.

Other patients required more complex plastic surgical procedures for fistula closure. Méndez-Fernández [8] described the use of a gastrocnemius myofascial flap and a split-thickness skin graft in two patients, after each had undergone two previous surgical treatments for knee fistulas that developed following surgical fixation of the tibia with Ender nails in one patient, and fixation of a comminuted patellar fracture in the other.

Jain and Varma [9] published a case of a knee fistula that developed after proximal tibial and patellar fractures. The tibial fracture had been treated with open reduction and internal fixation, whereas the patellar fracture was managed with patellectomy, and soft-tissue coverage was achieved using a split-thickness skin graft. Subsequently, the orthopedic hardware was removed because of infection associated with recurrent fistula formation. The patient then underwent saucerization of the fistula, coverage with a medial gastrocnemius muscle flap and split-thickness skin graft, followed four years later by exploration and direct closure of the recurrent fistula, with the knee immobilized in extension for two weeks. As the fistula recurred soon after mobilization was initiated, it was re-explored, its edges were debrided, and the resulting 1.1 cm defect in the knee joint capsule was repaired using a free radial artery fasciocutaneous flap.

## Conclusions

In conclusion, fistulas resulting from arthroscopic procedures appear to have a more benign course and may heal with immobilization alone. In contrast, fistulas resulting from open surgeries have the potential to become recurrent and may require more complex surgical procedures for definitive treatment. In our case, as the patient had previously undergone iliotibial band lengthening and lateral epicondyle osteotomy, and MRI had demonstrated multiple small openings without identifying a defined fistula, we chose to use a folded fascia lata allograft to reinforce the lateral and distal extra-articular portion of the patient's iliotibial tract. The graft was sutured with fibrin glue applied over the region surrounding the lateral epicondyle, a procedure that successfully resolved the condition in this patient.

Thus, the folded fascia lata allograft was successful in this patient and may represent a potential treatment option for selected cases of recurrent postsurgical knee synovial fistula, avoiding the need for a more invasive reconstructive procedure. However, further reports or comparative studies are needed before this technique can be generalized and applied in other cases.
